# Preoperative Anthropometric Parameters and Their Association With Five-Strand Hamstring Graft Diameter in Anterior Cruciate Ligament Reconstruction: A Retrospective Study From Southern Malaysia

**DOI:** 10.7759/cureus.110670

**Published:** 2026-06-11

**Authors:** Mohd Firdaus Abdullah, Zulkifli B Hassan, Nurul Huda Razali, Aisyah Ali, Munirah Ismail

**Affiliations:** 1 Orthopedic Surgery Department, Hospital Kuala Lumpur, Kuala Lumpur, MYS; 2 Trauma and Orthopedics Department, Hospital Sultan Ismail, Johor Bahru, MYS; 3 Clinical Research Centre, Hospital Sultan Ismail, Johor Bahru, MYS

**Keywords:** anterior cruciate ligament reconstruction, anthropometry, autograft, graft diameter, hamstring tendons, regression analysis

## Abstract

Background

Hamstring tendon (HT) autografts are commonly used in anterior cruciate ligament reconstruction (ACLR), although graft dimensions may vary among patients. Smaller graft diameters have been associated with higher graft failure rates, emphasizing the clinical importance of understanding factors related to the graft size. Consequently, preoperative anthropometric measurements have been investigated for their association with hamstring graft dimensions. However, findings from previous studies remain inconsistent across different populations, and data from Southeast Asian populations are still limited. Therefore, this study aimed to examine the relationship between preoperative anthropometric parameters and intraoperative hamstring graft dimensions, including diameter and length, in single-bundle ACLR patients using a five-strand hamstring autograft.

Methods

We retrospectively analyzed 91 patients who underwent primary single-bundle ACLR from 2018 to 2023 at a single institution. Preoperative data, including age, gender, height, weight, body mass index (BMI), and thigh circumference, were collected. Intraoperative graft diameter and length were obtained from the operative records. Pearson correlation and simple linear regression analyses were used to evaluate associations between anthropometric parameters and graft diameter. A p-value < 0.05 was considered statistically significant.

Results

Graft diameter showed a positive correlation with weight (r = 0.22, p = 0.040) and height (r = 0.51, p < 0.001), while graft length was positively correlated with height (r = 0.31, p = 0.003). Regression analysis confirmed a significant association between height and graft diameter (F (1, 89) = 30.649, p < 0.001), with height showing moderate explanatory power (R² = 0.256) and the strongest predictive relationship. In contrast, weight showed a statistically significant but weak association with graft diameter, explaining only a minimal proportion of variance (R² = 0.046).

Conclusion

Patient height showed a significant yet modest association with five-strand hamstring autograft diameter. These findings may provide supportive reference data for preoperative assessment in similar populations. Further studies are required to refine and validate predictive models for graft size estimation.

## Introduction

Anterior cruciate ligament reconstruction (ACLR) is a commonly performed orthopedic sports procedure, with an increasing incidence worldwide [1-4]. This surgery aims to recreate native functional stability in the ACL-deficient knee, particularly by resisting anteroposterior translation and rotational movement [1,3,4]. ACLR can be performed using various surgical techniques as well as graft materials. The hamstring tendon (HT) autograft is one of the most commonly used in ACLR since it was introduced in the 1980s [1]. Based on the latest global expert opinion survey, the majority of surgeons chose HT autograft as the primary graft during ACLR [2,3,5]. The semitendinosus tendon (ST), with or without the gracilis tendon (GT), is usually harvested on the ipsilateral leg as the donor graft to be used in ACLR. These grafts are commonly folded over each other to increase the diameter and fashioned into a quadruple (four-strand) graft. The quadruple graft gains its popularity due to excellent stiffness and tensile load properties, reduced donor site morbidity, improvements in fixation techniques and implants, better cosmesis, and excellent clinical outcomes [1,5].

Biomechanical studies have shown increased strength and stiffness of a hamstring graft with increased graft diameter. Hence, a graft diameter greater than 8 mm has been recommended by many authors [6-11]. The drawback of using a quadruple hamstring autograft is the likelihood of achieving a smaller graft diameter [6-9,11-13]. In the case of the undersized HT autograft, a five-strand graft technique can be adopted intraoperatively as a salvage procedure [14-17]. A five-strand hamstring autograft can be created by tripling the semitendinosus tendon and doubling the gracilis tendon. This technique has been reported to increase the graft diameter by 1-2 mm [14]. In our cohort, most of our patients who underwent an ACLR received a five-strand HT autograft prepared by a single surgeon. The surgical outcome between a quadruple- versus a five-strand HT autograft is comparable [15-17].

It is important to note that HT dimensions vary considerably among individuals, resulting in unpredictable intraoperative graft sizes. Therefore, several studies have investigated the relationship between HT autograft dimensions and patients’ anthropometric characteristics [6-9,11,18-23]. However, no general consensus has been reached due to inconsistent findings among these studies [18]. These discrepancies may be attributed to differences in geographic population characteristics, ethnicity, gender distribution, and activity levels across international cohorts. To the best of our knowledge, limited studies have evaluated the correlation between anthropometric measurements and HT graft dimensions in Southeast Asian populations. Therefore, this study aimed to evaluate the relationship between preoperative anthropometric variables and intraoperative HT autograft size in patients undergoing ACLR at our institution.

## Materials and methods

This study was conducted at Hospital Sultan Ismail, Johor Bahru, Malaysia, a tertiary referral center. It was a retrospective observational study involving patients who underwent arthroscopic single-bundle anterior cruciate ligament reconstruction (ACLR) using an autologous ipsilateral hamstring tendon (HT) autograft between January 2018 and December 2023. Data were retrieved from the hospital’s electronic medical record system (Health Information System, HIS Power Chart). Of 106 patients screened, 91 met the inclusion criteria and were included in the final analysis. Fifteen patients were excluded due to revision of ACLR using contralateral or allograft hamstring tendons, use of alternative graft types (e.g., a bone-patellar tendon-bone graft), or incomplete data involving more than 50% of study variables. The study was conducted in accordance with the Declaration of Helsinki, and ethical approval was obtained from the Medical Research Ethics Committee of the Ministry of Health Malaysia (NMRR ID-24-00979-HTI).

Surgical procedure

**Figure 1:**
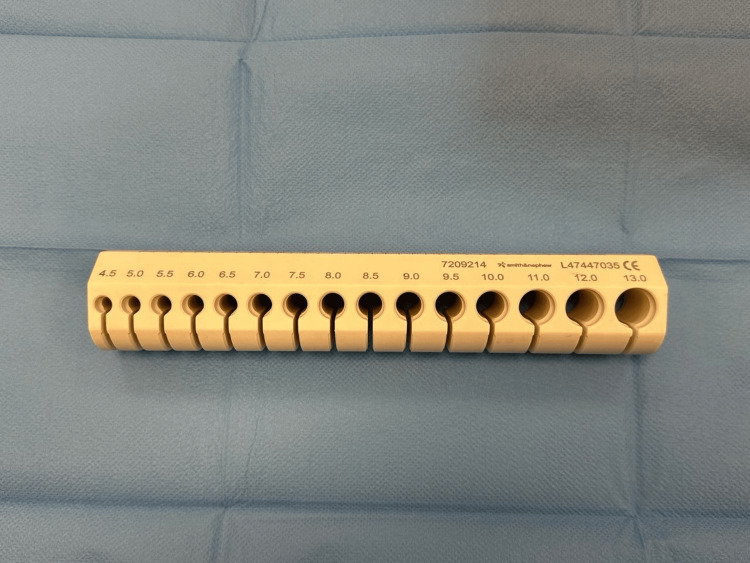
Graft diameter guide used for hamstring graft measurement during ACLR The instrument features sizing apertures with 0.5-mm increments. ACLR: anterior cruciate ligament reconstruction Photograph taken by the authors

All patients underwent arthroscopic single-bundle ACLR using an autologous hamstring tendon (HT) graft performed by a single surgeon. Both the semitendinosus (ST) and gracilis tendons (GT) were harvested using the standard manner. The harvested grafts were prepared in a single bundle, five-strand technique, with each end of the tendon grafts whipstitched with a No. 2 ETHIBOND EXCEL® Polyester Suture (Ethicon, Somerville, NJ). The final graft diameter was determined using the ACLR graft diameter measurement guide with 0.5 mm increments (Figure 1). The length of the HT graft was measured from the end-to-end length of the prepared graft. Femoral fixation was achieved using an Endo-Button, while tibial fixation was secured with a bioabsorbable interference screw. The final prepared HT graft diameter and length were documented in the operative records.

Study measurement

Sociodemographic and anthropometric data, including age, gender, ethnicity, height, weight, body mass index (BMI), and thigh circumference, were collected from the HIS PowerChart system. Thigh circumference was measured in a standardized manner 15 cm above the superior pole of the patella. Intraoperative graft data included the five-strand HT graft diameter (mm) and graft length (cm). All data were recorded using a proforma data collection form and entered into IBM SPSS Statistics version 27.0 (IBM Corp., Armonk, NY) for further analysis.

Statistical analysis

Statistical analysis was performed using the IBM SPSS Statistics for Windows version 27.0. Descriptive statistics were performed to summarize the sociodemographic and anthropometric variables of the patients. Mean and standard deviation (SD) were used to describe the demographic characteristics and anthropometric measurements of the patients for continuous data, whereas frequency and percentages (%) were used for categorical data.

Pearson’s correlation test was used to assess the relationship between anthropometric variables (age, height, weight, BMI, and thigh circumference) and graft diameter and length. The strength of the associations was interpreted according to the correlation coefficient. Then, variables that showed statistically significant associations in the bivariate analysis were subsequently entered into a linear regression model to examine the significance of the relationships. A p-value of less than 0.05 was considered statistically significant. Model assumptions were checked, including the normality of standardized residuals, and scatterplots of standardized residuals against predicted values were reviewed to assess model fit. Potential outliers and influential observations were also evaluated, and only models that satisfied the required assumptions were retained as the final models.

## Results

A total of 91 patients were included in the analysis, with a mean age of 27.7 years (ranging from 15 to 50 years). The study population was predominantly men, comprising 79 patients (86.8%), while women accounted for 12 patients (13.2%). Demographic and preoperative anthropometric measurements are summarized in Table 1.

**Table 1:** Demographic and preoperative anthropometric measurement data (N = 91) *Numerical data presented as mean (SD) for normally distributed data, and categorical data presented as frequency (percentage) SD: standard deviation, BMI: body mass index, Min-Max: minimum-maximum

Variables	Value*
Age (years)	
Mean ± SD	27.7 ± 8.6
Min-Max	15-52
Gender	
Male	79 (86.8)
Female	12 (13.2)
BMI (kg/m²)	
≤18.4	1 (1.1)
18.5-24.9	42 (46.2)
25.0-29.9	30 (33.0)
≥30.0	18 (19.8)
BMI (kg/m²)	
Mean ± SD	26.34 ± 4.82
Min-Max	17.4-38.7
Weight (kg)	
Mean ± SD	75.3 ± 14.8
Min-Max	50-116
Height (m)	
Mean ± SD	1.69 ± 0.07
Min-Max	1.52-1.85
Thigh circumference (cm)	
Mean ± SD	54.0 ± 6.0
Min-Max	41-70
Graft diameter (mm)	
Mean ± SD	8.58 ± 0.67
Min-Max	7-10
Graft length (cm)	
Mean ± SD	9.09 ± 0.61
Min-Max	8-10

The mean patient height was 1.69 ± 0.07 m, and the mean weight was 75.28 ± 14.80 kg. Regarding BMI, 42 patients (46.2%) were in the normal range, 30 (33.0%) were overweight, 18 (19.8%) were obese, and 1 patient (1.1%) had a BMI below normal. The hamstring graft diameter ranged from 7.0 to 10.0 mm, while graft length ranged from 8.0 to 10.0 cm.

**Figure 2:**
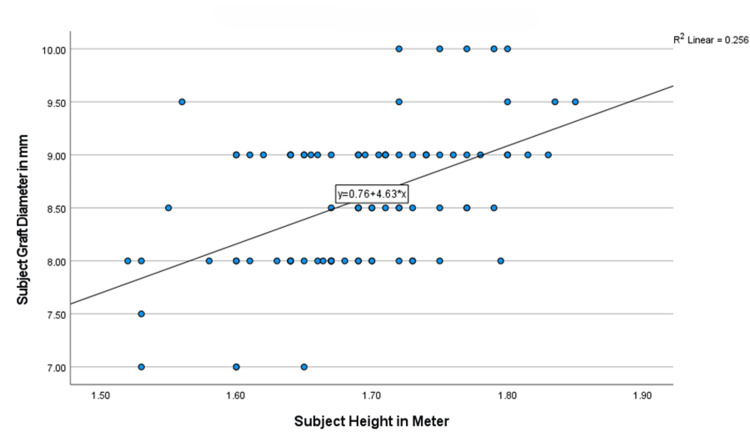
Scatterplots showing correlation between height and hamstring graft diameter (N = 91) *Pearson’s correlation test

**Figure 3:**
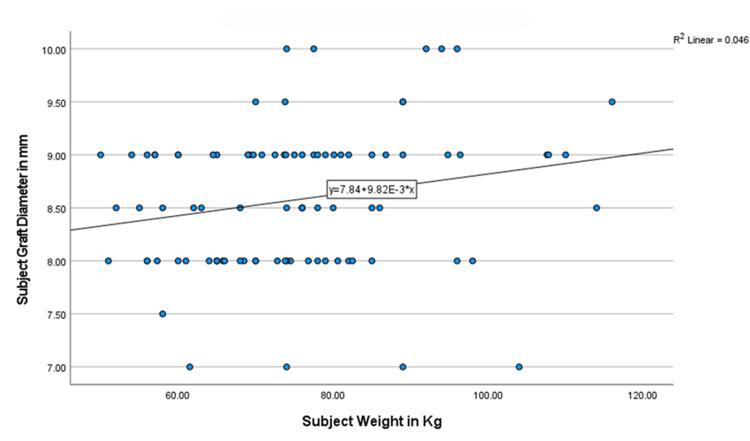
Scatterplots showing correlation between weight and hamstring graft diameter (N = 91) *Pearson’s correlation test

**Figure 4:**
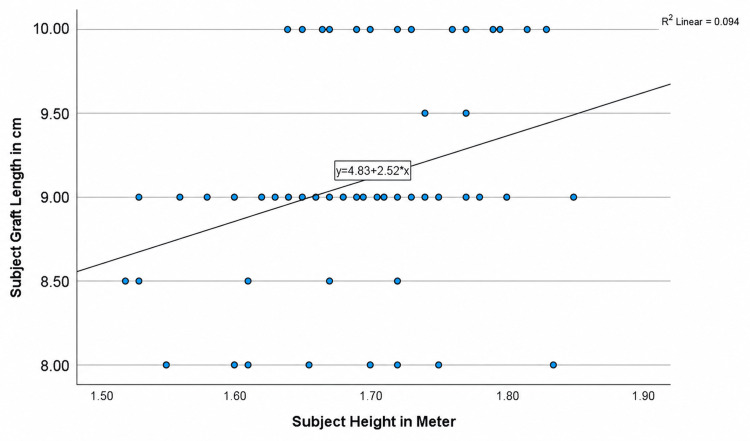
Scatterplots showing correlation between height and hamstring graft length (N = 91) *Pearson’s correlation test

Pearson’s correlation analysis revealed that patient height and weight were positively associated with graft diameter (Figure 2 and Figure 3, respectively). Height showed a moderate correlation with graft diameter (r = 0.51, n = 91, p < 0.001), whereas weight had a weak correlation (r = 0.22, n = 91, p = 0.040). Other anthropometric variables demonstrated poor or no significant correlation with graft diameter (Table 2). For the graft length, only height showed a moderate correlation (r = 0.31, n = 91, p = 0.003) (Table 2, Figure 4).

**Table 2:** Correlation between graft diameter and length with anthropometric variables (N = 91) r = Pearson’s correlation coefficient BMI: body mass index

Variable	Graft diameter		Graft length	
	r	p-value	r	p-value
Age (years)	-0.112	0.290	0.010	0.925
Weight (kg)	0.216	0.040	0.099	0.348
Height (m)	0.506	<0.001	0.306	0.003
BMI (kg/m²)	-0.028	0.759	-0.038	0.719
Thigh circumference (cm)	-0.027	0.798	0.046	0.663

Subsequently, simple linear regression analysis was performed to determine the influence of the preoperative anthropometric variables on the intraoperative graft measurement. For graft diameter, there was a statistically significant linear relationship between patient height and the graft diameter (F (1, 89) = 30.649, p < 0.001), with height explaining 25.6% of the variance in graft size (R² = 0.256). Weight demonstrated a weak but statistically significant association with graft diameter (F(1,89) = 4.335, p < 0.040), accounting for 4.6% of the variance (R² = 0.046). In contrast, age, BMI, and thigh circumference were not significantly associated with graft diameter (Table 3). Based on these findings, a stepwise regression analysis was performed, which confirmed height as the only variable retained in the final model. The regression equation for graft diameter was 0.759 + 4.625 (height), where graft diameter is measured in millimeters and height in meters. Hence, participants’ graft diameter increased by approximately 0.46 mm for each 10 cm increase in height.

**Table 3:** Simple linear regression analysis between graft diameter and anthropometric variables (N = 91) ß: regression coefficient, CI: confidence interval, SE: standard error, R²: coefficient of determination, BMI: body mass index

Variable	Graft diameter					
	ß (95% CI)	Intercept	SE	R^2^	F	p-value
Age (years)	-0.009 (-0.025, 0.008)	8.820	0.008	0.013	1.132	0.290
Weight (kg)	0.010 (0.000, 0.019)	7.837	0.005	0.046	4.335	0.040
Height (m)	4.625 (2.965, 6.285)	0.759	0.835	0.256	30.649	<0.001
BMI (kg/m²)	-0.004 (-0.033, 0.026)	8.679	0.015	0.001	0.068	0.795
Thigh circumference (cm)	-0.003 (-0.027, 0.021)	8.741	0.012	0.001	0.066	0.798

A secondary analysis was performed for graft length. Height showed a statistically significant association with graft length (F (1, 89) = 9.198, p = 0.003), explaining 9.4% of the variance (R² = 0.094). The stepwise regression analysis confirmed height as the only variable retained in the model. Graft length increased by approximately 2.522 cm for each 1-m increase in height.

## Discussion

The hamstring tendon (HT) autograft is the most commonly used graft for primary ACLR at our institution, owing to its well-established advantages over other graft types [1,5,24]. Despite these benefits, inadequate graft size remains a recognized concern, as smaller graft diameters have been associated with higher failure and revision rates [6,7,13]. For this reason, being able to estimate graft dimensions preoperatively is clinically important. To date, no study has examined the relationship between preoperative anthropometric parameters and HT autograft dimensions in the Malaysian population, particularly in the southern region. This study aimed to explore this relationship within our local cohort.

The clinical importance of graft diameter has been well described in the literature. Magnussen et al. reported that hamstring autografts ≤ 8 mm were associated with higher revision rates [7]. This was further supported by Conte et al. [6] and registry analyses including 18,425 patients from Sweden and Norway [24], which showed increased revision risk for grafts <8 mm. Some studies have suggested that a graft diameter as small as 7 mm is still acceptable [13,25]. Nevertheless, graft diameters of ≤6.5 mm are associated with a higher risk of failure [25]. In our series, only five patients (two male and three female patients) had graft diameters below 8 mm, with the smallest measuring 7 mm, and none required revision prior to this publication. While this finding is reassuring, the absence of revision cannot be attributed solely to graft size, as other factors may have contributed and warrant further investigation.

In the present study, height showed a significant positive association with graft diameter, with approximately 25.6% of the observed variability explained (R² = 0.256). Meanwhile, weight demonstrated a weak positive association in the initial analysis but was not retained as an independent variable in the final regression model. These findings indicate that although taller individuals tend to have larger hamstring tendon graft diameters, height alone accounts for only a moderate proportion of the variability, suggesting the influence of additional anatomical and patient-specific factors. Based on the literature, various authors have reported significant associations between height, weight, and graft diameter [6-9,12,19,22,24,26-29]. Singhal et al., in a study involving 280 patients, similarly reported moderate positive correlations between height and weight, and graft size (R = 0.64 and R = 0.47, respectively) [27]. Likewise, Pinheiro et al., in a prospective study of 80 patients, demonstrated that graft diameter was significantly associated with height and weight (R = 0.47 and R = 0.36, respectively), as well as leg length, thigh length, thigh diameter, and male gender [28]. Similarly, Çeliktaş et al., in a cohort of 164 Turkish patients, also reported positive correlations between height (R = 0.397) and weight (R = 0.245) and graft diameter [9]. Collectively, these findings support the relevance of anthropometric parameters, particularly height, in relation to graft size.

The explanatory value of anthropometric-based models for graft diameter has varied across published studies. In the present study, height accounted for 25.6% of the variability in graft diameter (R² = 0.256), which is comparable to findings reported in other populations. Çeliktaş et al. [9] reported a lower coefficient of determination (R² = 0.157) in a Turkish cohort, while Ho et al. [11] demonstrated a higher explanatory value (R² = 0.358) in a Singaporean population. In contrast, Tuman et al. [29] reported a relatively weak association in a United States cohort (R² = 0.13), whereas Asif et al. [26] observed a substantially stronger relationship in an Indian population (R² = 0.80). These variations may reflect differences in geographical factors, ethnicity, body habitus, sample size, graft preparation techniques, and measurement methods across studies. Collectively, these findings suggest that while height demonstrates a consistent association with hamstring graft diameter, the strength of this association is population dependent and remains insufficient for precise individual estimation. Nevertheless, the moderate coefficient of determination observed in the present study suggests that graft diameter is likely influenced by additional anatomical and patient-related factors that warrant further investigation.

Although patient height demonstrated a positive association with final graft length in our cohort, the correlation was weak, suggesting that height alone contributes only minimally to graft length variability and that other anthropometric or anatomical factors may play a more important role. These findings contrast with previous studies. Gupta et al. reported strong correlations between height and semitendinosus tendon length (r = 0.719), gracilis tendon length (r = 0.768), and final graft diameter (r = 0.685) [19]. Similarly, Dietvorst et al. demonstrated that height was positively associated with tendon length in adolescents, with both semitendinosus and gracilis tendons increasing with increasing height [22]. They further noted that adequate semitendinosus length could achieve sufficient graft diameter in most cases, while gracilis augmentation was more frequently required in shorter patients.

In the present study, individual semitendinosus and gracilis tendon lengths were not routinely recorded, as only the final prepared graft length was measured intraoperatively. This may have limited a more precise assessment of the relationship between anthropometric variables and tendon morphology, as final graft length may also be influenced by harvesting and preparation techniques. Nevertheless, the minimum graft length observed (8 cm) was sufficient for standard tibial fixation using an interference screw. Preoperative estimation of graft length remains clinically relevant, as inadequate graft length may compromise fixation, particularly on the tibial side [11,16,29]. In cases where graft diameter is adequate but length is limited (<8 cm), suspensory fixation on both femoral and tibial sides may be considered as an alternative strategy [30].

Our analysis also demonstrated no significant correlation between age and BMI, and graft diameter, findings that are consistent with previous literature. Atbaşi et al. similarly reported no significant correlation between BMI and quadrupled hamstring graft size [21]. Furthermore, a systematic review by Salman et al. found that the pooled correlation between age and graft diameter was negligible (COR: 0.02; 95% CI: -0.03 to 0.06; p = 0.462), while BMI demonstrated only a weak association with graft diameter (COR: 0.17; 95% CI: 0.11-0.23; p < 0.001) [18].

It is important to note that most previously published studies evaluating anthropometric factors associated with graft size were based on quadrupled hamstring graft configurations. In contrast, our study specifically evaluated a five-strand HT construct, reflecting the routine surgical practice at our institution during the study period. The majority of patients had complete operative documentation for the five-strand configuration, whereas records for four-strand grafts were fewer and often incomplete. For consistency and data reliability, only patients with complete five-strand graft data were included in the analysis. Despite the difference in graft configuration, patient height showed a significant positive association with graft diameter in our cohort, consistent with trends reported in studies involving four-strand hamstring grafts [9,11,18-22,26-29].

Although our findings generally align with existing international literature, this study also provides valuable data within the Southeast Asian population, where region-specific evidence remains limited. Anthropometric characteristics may vary substantially across different ethnic populations, and regression models derived predominantly from Western cohorts may not be directly applicable to Southeast Asian patients. Our findings, therefore, offer useful local reference data that may aid preoperative counseling, graft planning, and intraoperative decision-making during ACLR.

Several limitations should be acknowledged. This was a retrospective single-center study with a relatively small sample size, which may limit generalizability. The cohort may not fully represent the anthropometric diversity of the Malaysian population. In addition, only final prepared graft dimensions were recorded, whereas individual semitendinosus and gracilis tendon measurements were not routinely documented, thereby limiting more detailed tendon-specific analysis. There was also a higher proportion of male participants compared with female patients. Therefore, the findings may not be fully generalizable to the female population. Future studies with larger cohorts and more comprehensive data collection, including individual tendon measurements, graft configuration details, and additional potential confounders such as activity level, limb dominance, and ethnic background, may provide a more robust understanding of factors influencing graft dimensions.

Further research incorporating multivariate regression modeling may also help develop more reliable predictive models for hamstring graft sizing in ACLR. In addition, integrating preoperative imaging modalities such as magnetic resonance imaging or ultrasound may improve the accuracy of graft size estimation and enhance surgical planning. Collectively, these approaches may contribute to a comprehensive understanding of factors influencing graft adequacy and ultimately optimize outcomes following ACLR.

## Conclusions

We concluded that patient height is an important predictor of graft diameter in ACLR, with a significant association observed for the five-strand hamstring tendon autograft configuration in our setting. Although the predictive model showed moderate explanatory capability, these findings suggest that graft dimensions are likely influenced by multiple factors beyond anthropometric measurements and may limit generalizability. This study contributes valuable regional data on the association between anthropometric parameters and graft diameter in ACLR. Further prospective studies with larger sample sizes and the inclusion of additional anatomical and clinical variables may improve the accuracy of preoperative graft estimation models.

## References

[1] Shaerf DA, Pastides PS, Sarraf KM, Willis-Owen CA (2014). Anterior cruciate ligament reconstruction best practice: a review of graft choice. World J Orthop.

[2] Sherman SL, Calcei J, Ray T (2021). ACL Study Group presents the global trends in ACL reconstruction: biennial survey of the ACL Study Group. J ISAKOS.

[3] Tuca M, Valderrama I, Eriksson K, Tapasvi S (2023). Current trends in anterior cruciate ligament surgery. A worldwide benchmark study. J ISAKOS.

[4] Kaeding CC, Léger-St-Jean B, Magnussen RA (2017). Epidemiology and diagnosis of anterior cruciate ligament injuries. Clin Sports Med.

[5] Figueroa F, Figueroa D, Espregueira-Mendes J (2018). Hamstring autograft size importance in anterior cruciate ligament repair surgery. EFORT Open Rev.

[6] Conte EJ, Hyatt AE, Gatt CJ Jr, Dhawan A (2014). Hamstring autograft size can be predicted and is a potential risk factor for anterior cruciate ligament reconstruction failure. Arthroscopy.

[7] Magnussen RA, Lawrence JT, West RL, Toth AP, Taylor DC, Garrett WE (2012). Graft size and patient age are predictors of early revision after anterior cruciate ligament reconstruction with hamstring autograft. Arthroscopy.

[8] Goyal S, Matias N, Pandey V, Acharya K (2016). Are pre-operative anthropometric parameters helpful in predicting length and thickness of quadrupled hamstring graft for ACL reconstruction in adults? A prospective study and literature review. Int Orthop.

[9] Çeliktaş M, Gölpinar A, Köse Ö, Sütoluk Z, Çelebi K, Sarpel Y (2013). Prediction of the quadruple hamstring autograft thickness in ACL reconstruction using anthropometric measures. Acta Orthop Traumatol Turc.

[10] Mirzayan R, Chang RN, Royse KE, Reyes CE, Prentice HA, Maletis GB (2025). Is there a hamstring autograft diameter threshold for anterior cruciate ligament reconstruction?. Orthop J Sports Med.

[11] Ho SW, Tan TJ, Lee KT (2016). Role of anthropometric data in the prediction of 4-stranded hamstring graft size in anterior cruciate ligament reconstruction. Acta Orthop Belg.

[12] Park SY, Oh H, Park S, Lee JH, Lee SH, Yoon KH (2013). Factors predicting hamstring tendon autograft diameters and resulting failure rates after anterior cruciate ligament reconstruction. Knee Surg Sports Traumatol Arthrosc.

[13] Alomar AZ, Nasser AS, Kumar A, Kumar M, Das S, Mittal S (2022). Hamstring graft diameter above 7 mm has a lower risk of failure following anterior cruciate ligament reconstruction. Knee Surg Sports Traumatol Arthrosc.

[14] Lee RJ, Ganley TJ (2014). The 5-strand hamstring graft in anterior cruciate ligament reconstruction. Arthrosc Tech.

[15] Krishna L, Tan XY, Wong FK, Toh SJ (2018). A 5-strand hamstring autograft achieves outcomes comparable to those of a 4-strand hamstring autograft with a graft diameter of 8 mm or more in anterior cruciate ligament reconstruction. Orthop J Sports Med.

[16] Calvo R, Figueroa D, Figueroa F, Vaisman A, Schmidt-Hebbel A, Morales N, Izquierdo G (2017). Five-strand hamstring autograft versus quadruple hamstring autograft with graft diameters 8.0 millimeters or more in anterior cruciate ligament reconstruction: clinical outcomes with a minimum 2-year follow-up. Arthroscopy.

[17] Smith JH, Houck DA, Hart JA, Vidal AF, Frank RM, Bravman JT, McCarty EC (2019). Five-strand hamstring autografts for anterior cruciate ligament reconstruction: a systematic review. Orthop J Sports Med.

[18] Salman LA, Moghamis IS, Hatnouly AT (2024). Correlation between anthropometric measurements and graft size in anterior cruciate ligament reconstruction: a systematic review and meta-analysis. Eur J Orthop Surg Traumatol.

[19] Gupta R, Malhotra A, Masih GD, Khanna T (2017). Equation-based precise prediction of length of hamstring tendons and quadrupled graft diameter by various anthropometric variables for knee ligament reconstruction in Indian population. J Orthop Surg (Hong Kong).

[20] Janssen RP, van der Velden MJ, van den Besselaar M, Reijman M (2017). Prediction of length and diameter of hamstring tendon autografts for knee ligament surgery in Caucasians. Knee Surg Sports Traumatol Arthrosc.

[21] Atbaşi Z, Erçin E, Erdem Y, Emre TY, Atilla HA, Parlak A (2016). Correlation between body mass index and quadrupled hamstring tendon autograft size in ACL reconstruction. Joints.

[22] Dietvorst M, van der Steen MC, van den Besselaar M, Janssen RP (2023). Height is a predictor of hamstring tendon length and ACL graft characteristics in adolescents. BMC Musculoskelet Disord.

[23] Sarakatsianos V, Cristiani R, Forssblad M, Edman G, Stålman A (2024). Patient’s height and sex predict graft diameter: a cohort study of 4,519 patients with primary anterior cruciate ligament reconstruction using semitendinosus autograft. Arthroscopy.

[24] Snaebjörnsson T, Hamrin-Senorski E, Svantesson E, Karlsson L, Engebretsen L, Karlsson J, Samuelsson K (2019). Graft diameter and graft type as predictors of anterior cruciate ligament revision: a cohort study including 18,425 patients from the Swedish and Norwegian national knee ligament registries. J Bone Joint Surg Am.

[25] Kang H, Dong C, Wang F (2019). Small hamstring autograft is defined by a cut-off diameter of 7 mm and not recommended with allograft augmentation in single-bundle ACL reconstruction. Knee Surg Sports Traumatol Arthrosc.

[26] Asif N, Ranjan R, Ahmed S, Sabir AB, Jilani LZ, Qureshi OA (2016). Prediction of quadruple hamstring graft diameter for anterior cruciate ligament reconstruction by anthropometric measurements. Indian J Orthop.

[27] Singhal D, Kanodia N, Singh R, Singh SK, Agrawal S (2021). Predicting quadruple semitendinosus graft size for anterior cruciate ligament reconstruction by patient anthropometric variables: a cohort study of 280 cases. Malays Orthop J.

[28] Pinheiro LF Jr, de Andrade MA, Teixeira LE (2011). Intra-operative four-stranded hamstring tendon graft diameter evaluation. Knee Surg Sports Traumatol Arthrosc.

[29] Tuman JM, Diduch DR, Rubino LJ, Baumfeld JA, Nguyen HS, Hart JM (2007). Predictors for hamstring graft diameter in anterior cruciate ligament reconstruction. Am J Sports Med.

[30] Fritsch B, Figueroa F, Semay B (2017). Graft preparation technique to optimize hamstring graft diameter for anterior cruciate ligament reconstruction. Arthrosc Tech.

